# Long Noncoding RNA KCNMB2-AS1 Promotes SMAD5 by Targeting miR-3194-3p to Induce Bladder Cancer Progression

**DOI:** 10.3389/fonc.2021.649778

**Published:** 2021-05-07

**Authors:** Yong-Sheng Chen, Yong-Peng Xu, Wen-Hua Liu, De-Chao Li, Huan Wang, Chang-Fu Li

**Affiliations:** ^1^ Department of Urology, Harbin Medical University Cancer Hospital, Harbin, China; ^2^ Intensive Care Unit (ICU) Department, The Second Affiliated Hospital of Harbin Medical University, Harbin, China

**Keywords:** bladder cancer, KCNMB2-AS1, miR-3194-3p, stemness, SAMD5

## Abstract

**Purpose:**

Bladder cancer is a common malignant tumor of the urinary system, with the fourth-highest incidence of male malignant tumors in Europe and the United States. So far, the mechanism of bladder cancer progression and metastasis has not been clarified. The aim of our study was to validate the way of long noncoding RNA (lncRNA) KCNMB2-AS1 on the metabolism and growth of bladder cancer cells by miR-3194-3p/SMAD5.

**Patients and Methods:**

The Gene Expression was analyzed by qRT-PCR in bladder cancer tissues and cell lines, with the highly expressed KCNMB2-AS1 screened out. Cell proliferation was detected by Edu staining and clone formation assay, cell migration, and invasion by wound healing and transwell assays. Cell stemness was determined by assessing sphere-forming ability and stemness marker. Correlation between miRNA and lncRNA/gene was verified by dual‐luciferase assay and RIP, and the effect of KCNMB2-AS1 on bladder cancer growth by nude mice tumor formation experiment.

**Results:**

Here, we revealed the increased level of KCNMB2-AS1 in bladder cancer for the first time. Knockdown of KCNMB2-AS1 *in vitro* prevented the ability of proliferation, metastasis, and stemness of cancer cells. *In vivo*, the silencing of KCNMB2-AS1 also prevented tumor growth *in vivo*. Next, we revealed that KCNMB2-AS1 could interact with miR-3194-3p and uncovered that SAMD5 was a downstream target of miR-3194-3p.

**Conclusion:**

In conclusion, KCNMB2-AS1 mediated the bladder cancer cells progress by regulating the miR-3194-3p/SAMD5 signal pathway, which would provide a new target for bladder cancer research.

## Introduction

The incidence of bladder cancer is higher in urinary system tumors, and 150,000 people die from bladder cancer every year (1, 2). In China, the incidence is about 7.3/100,000 in males and 2/100,000 in females (3), which is increasing and poses a threat to people’s health. The occurrence of bladder cancer has many eccentricities and a high recurrence rate (4). Patients need regular reexamination and long-term monitoring, which is a heavy burden on families and society. Bladder cancer has received more and more attention in recent years. The pathogenesis and mechanism of bladder cancer still need further study.

LncRNA is a non-coding RNA that is more than 200 bp long and can regulate gene expression. LncRNA would affect different cellular functions, which would cause changes in molecular functions of related signal pathways and change cell life activities. As an important form of epigenetics, lncRNA plays an important role at different levels of cells. Abnormal expression of specific lncRNA has been found in kinds of tumors, and this abnormality often suggests that lncRNA is related to the biological process of tumorigenesis (5, 6). LncRNA promotes urothelial cell proliferation and inhibits apoptosis through the molecular signaling pathway, which leads to the transformation of malignant degree. Therefore, lncRNA regulated progress of bladder cancer may become a biomarker for the diagnosis and prognosis of bladder cancer (7). Previous studies reported that the expression of lncRNA upregulated gene factor 1 (UBC1) in bladder cancer tissue was up-regulated by microarray and qRT-PCR technology, and the siRNA technique found that knockout UBC1 could inhibit the metastasis of bladder cancer cells (8, 9). It has been found that the expression of lncRNA GAS5 is decreased in bladder cancer specimens, and overexpression of lncRNA GAS5 prevents the proliferation and migration of bladder cancer cells (10, 11). Previous research has found that the level of lncRNA H19 is up-regulated in bladder cancer and then promotes the metastasis of bladder cancer by inducing epithelial–mesenchymal transformation, thus promoting the occurrence and development of bladder cancer (12). Analysis of bladder cancer tissue showed that LncRNA-n346372 was increased in bladder cancer tissues (13). Early studies have reported that lncRNA UCAl, MEG3, and PVT1 may be molecular markers for the diagnosis of bladder cancer (14, 15). The expression level of MALAT1 is the expression in bladder cancer and is related to tumor metastasis and grade (16). The expression of lncRNA ROR and lncRNA ATB in the serum of patients with bladder cancer is significantly up-regulated. Meanwhile, the high expression of lncRNA ROR and ATB is correlated with prognostic indicators, which may be participated in the occurrence and development of bladder cancer (17, 18). In addition, lncRNA HOTAIR was significantly up-regulated in patients with 81.8% T1 and T2 bladder cancer, and the up-regulated lncRNA HOTAIR was closely related to bladder cancer recurrence and cell infiltration (19, 20). Studies have found that many abnormally expressed lncRNAs are related to tumor staging and survival, so these abnormally expressed lncRNAs may be potential prognostic indicators of bladder cancer.

So far, the function of KCNMB2-AS1 in bladder cancer was unclear and needed investigation. In this research, we explored the functional role of KCNMB2-AS1 and revealed the potential pathway in bladder cancer.

## Methods and Materials

### Clinical Samples

The tumor samples and adjacent normal samples were collected from 56 bladder cancer patients at Harbin Medical University Cancer Hospital. All of the patients or their guardians provided written consent. This study was reviewed and approved by the Ethics Committee of Harbin Medical University Cancer Hospital and was conducted according to the international guidelines of the Helsinki Declaration.

### Cell Culture

SV-HUC-1 (Human bladder epithelial immortalized cells), EJ, BIU87, 5637, and T24 (Human bladder cancer cells) cell lines were purchased from the Science Cell Laboratory. SV-HUC-1 cells were cultured in F12K with 10% FBS and 100 μl/ml penicillin and streptomycin (Beyotime, China) and placed at 37°C with 5% CO_2_. EJ cells were cultured in MEM-EBSS with 10% FBS and 100 μl/ml penicillin and streptomycin (Beyotime, China) and placed at 37 °C with 5% CO_2_. BIU87 and 5637 cells were cultured in RPMI-1640 with 10% FBS and 100 μl/ml penicillin and streptomycin (Beyotime, China) and placed at 37°C with 5% CO_2_. T24 cells were cultured in McCoy’s 5A with 10% FBS and 100 μl/ml penicillin and streptomycin (Beyotime, China) and placed at 37°C with 5% CO2.

### Cell Transfection

Sh-lncRNA was produced by Ribobio Co., Ltd. (Guangdong, China). Sh-NC (negative control) was indicated as control (sh-NC). Cells were cultured in 6-well plates; Until 70–80% of confluence, cells were transfected using Lipofectamine 2000 (Invitrogen, Carlsbad, CA, USA). Briefly, plasmid or si-RNA was diluted in Opti-MEM, stand for 5 min, and mixed with RNA/DNA-Lipofectamine 2000. Transfection solution stood for 20 min and applied in each well. Transfected cells were harvested at 48 h for the following experiments.

### Quantitative Real-Time PCR

Total RNA was extracted from bladder cancer cells using TRIzol reagent (Invitrogen, USA), and their relative complementary deoxyribose nucleic acid (cDNA) was synthesized by Primescript RT Reagent (TaKaRa, Tokyo, Japan). QRT-PCR was performed by using StepOne Plus Real-Time PCR system (Applied Biosystems, USA) with SYBR^®^Premix Ex Taq™ Reagent (TaKaRa, Japan). QRT-PCR was performed at 95°C for 5 min, 95°C for 15 s, 58°C for 30 s and 74°C for 30 s, for a total of 40 cycles. Gene expression was calculated using the 2^−ΔΔCt^ method. The KCNMB2-AS1 primers sequences: 5′- GCAGTTTGATCTCAGACTGCTGTG−3′ (forward) and 5′- TTTATTTCCTGTAGTCTCAGCTACTCAG-3′ (reverse).

### Western Blot

Total protein was collected from tumor tissues or cell lines with RIPA lysis Mix. The western blotting assay was performed as previously described. Briefly, 50–80 μg protein extraction was loaded *via* SDS-PAGE and transferred onto nitrocellulose membranes (Absin, China), then incubated with primary antibodies, anti-CD133 (1:2,000, ab222782), anti-Nanog (1:1,000, ab109250), anti-Oct14(1:10,000, ab200834), anti-Sox2(1:1,000, ab97959), anti-ALDH1 (1:1,000, ab56777), anti-GAPDH (1:2,000, ab8245) was used as the loading control for 2 h at temperature, then plated at 4°C overnight, the membranes were incubated in 5% non-fat milk blocking buffer for horizontal mode 3 h. After incubation with secondary antibodies, the membranes were scanned using an Odyssey, and data were analyzed with Odyssey software (LI-COR, USA).

### Fluorescence *In Situ* Hybridization (FISH)

The FISH assay was performed in 5637 cells according to the specifications of the manufacturers. The Cy3-labeled KCNMB2-AS1 probes used in our study were designed and synthesized by GenePharma (Shanghai, China). Briefly, the prepared cells were fixed with 4% paraformaldehyde for 30 min. After permeabilization, the cells were incubated with specific probes at 37°C overnight. The cell nuclei were stained with DAPI (Sigma-Aldrich, USA). The staining results were observed using a fluorescence microscope (Nikon, Japan).

### Matrigel Invasion Assay

BIU87 and 5637 cells in the logarithmic growth phase were adjusted to 2 × 10^5^ cells/well of medium (without serum) and plated 1 μg/μl Matrigel into the upper chamber. The lower chamber was added with 500 μl of the medium, and then incubate the plate at 37°C for 48 h. Then the invading cells were visualized by the crystal violet and inverted microscope.

### *In Vivo* Tumor Growth Assay

Nude mice were purchased from the Beijing Charles river. sh-KCNMB2-AS1-5637/sh-NC-5637 cells were subcutaneously injected in the right lower limb of the nude mice. Tumor size was measured every five days. After another 15 d of injection, mice were intraperitoneally injected with 3% pentobarbital sodium and were euthanatized by excessive anesthesia with a dose of 90 ml/kg, and the tumors were removed for follow-up study. The animal study was reviewed and approved by Harbin Medical University Cancer Hospital.

### Tumor Sphere Formation Assay

The cells in good growth condition were digested and centrifuged. The serum-containing medium was removed and washed with PBS 2 times. Cells were resuspended and counted in stem cell medium (DMEM/F12 + 1XB27 + 20 ng/ml bFGF + 20 ng/mI EGF). Ultralow adsorption cells were selected to culture six well plates, N cells per well (500 cells), supplemented with 4 ml medium. About 10 days to complete the culture, observe the ball state.

### Flow Cytometry Assay

The treated bladder cancer cells were first incubated with Hoechst 33342 (Invitrogen) for 90 min at 37 °C. After washing with PBS, cells were trypsinized and filtered, and resuspended in ice-cold PBS. Propidium iodide was then added 5 min before analysis. Cells were analyzed by using a DakoCytomation MoFlo cytometer (Beckham Coulter, 9 Brea, USA) using dual-wavelength analysis (red, 670 nm, blue, 450 nm).

### Luciferase Assay

20 mmol/L miRNA mimic or miR-NC and lncRNA-WT or lncRNA-mutation were co-transfected into HEK293T cells. Luciferase activity was detected with Luciferase Reporter Assay Kit (Biovision, China) on a luminometer (Berthold, Germany) 48 h after the transfection.

### Statistical Analysis

All values are calculated as the mean ± SEM. Statistical significances were measured by Student’s t-test and ANOVA. The correlation among relative KCNMB2-AS1, miR-3194-3p and SMAD5 expression in bladder cancer tissues was determined by Spearman’s correlation test. A two-tailed value of *P* < 0.05 was indicated as a statistically significant difference. Data statistics were used the GraphPad 7.0.

## Results

### The Increased Level of KCNMB2-AS1 in Bladder Cancer

**Figure 1:**
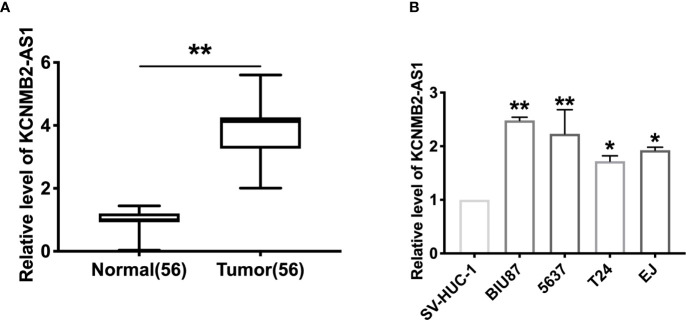
The increased expression of KCNMB2-AS1 in bladder cancer. **(A)** KCNMB2-AS1 expression in 56 pairs of bladder cancer and adjacent normal tissues detected by qRT-PCR. **(B)** The expression of KCNMB2-AS1 in bladder cancer cell lines and the normal SV-HUC-1 cell line. n = 7. *P < 0.05, **P < 0.01.

To explore the function of KCNMB2-AS1 in bladder cancer, we collected the 56 bladder cancer patients’ tumor tissues and paired normal adjacent bladder tissues. RT-PCR was employed to detect the expression of KCNMB2-AS1. We observed that the level of KCNMB2-AS1 was up-regulated in tumor tissues (**Figure 1A**). Then we detected the expression of KCNMB2-AS1 in four different human bladder cancer cell lines (EJ, BIU87, 5637, and T24), and SV-HUC-1 was described as control. Compared with SV-HUC-1 cells, the expression level of KCNMB2-AS1 was significantly up-regulated in EJ, BIU87, 5637, and T24 cells (**Figure 1B**).

### Silencing of KCNMB2-AS1 Remitted Bladder Cancer Cell Proliferation, Migration, and Invasion

**Figure 2:**
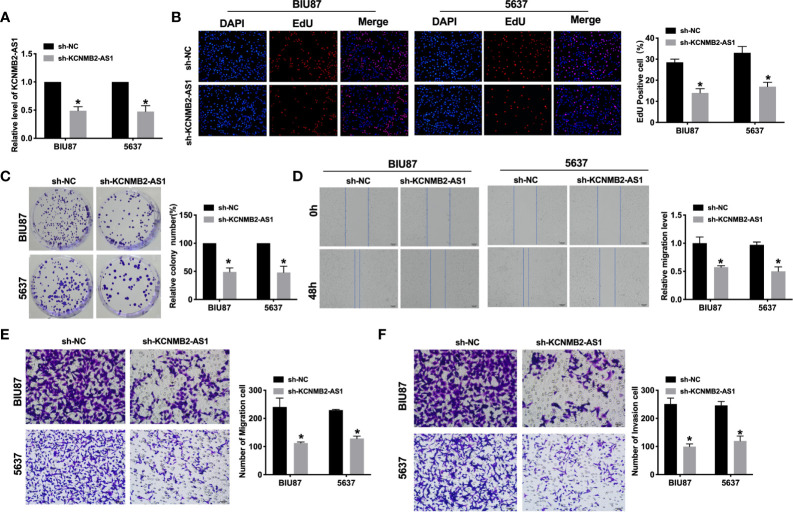
Inhibition of KCNMB2-AS1 attenuated bladder cancer cell proliferation, migration, and invasion. **(A)** Transfection efficiency was detected by RT-PCR. n = 10. **(B)** EdU incorporation of cancer cells was used to measure cell proliferation rate. n = 8. **(C)** Clone formation capacity of BIU87 and 5637 cells was assessed by the clone formation assay. n = 5. **(D)** The typical pictures of Scratch-wound healing assay. **(E)** Tumor cell migration study *via* transwell assay. n = 6. **(F)** Detecting cell invasion by Transwell invasion assay. n = 6. *P < 0.05.

Further, we constructed the shRNA for knockdown the expression of KCNMB2-AS1 (sh- KCNMB2-AS1), and sh-NC was indicated as a negative control. BIU87 and 5637 cells were transfected with sh-KCNMB2-AS1/sh-NC for 48 h. The transfection efficiency was detected by RT-PCR (**Figure 2A**). As shown in **Figure 2B**, downregulation of KCNMB2-AS1 inhibited the proliferation of BIU87 and 5637 cells, which was indicated by the Edu assay. Clone formation assay highlighted the evidently reduced proliferation of BIU87 and 5637 cell lines treated with sh-KCNMB2-AS1 (**Figure 2C**). Wound healing assays showed that knockdown of KCNMB2-AS1 prevented the migration of BIU87 and 5637 cells (**Figure 2D**). Transwell assays also showed similar results (**Figure 2E**). We observed that sh-KCNMB2-AS1 inhibited the invasion of bladder cancer cells by performing a matrigel invasion assay (**Figure 2F**). In summary, knockdown of KCNMB2-AS1 would inhibit the ability of proliferation and metastasis in bladder cancer cell lines.

### Knockdown of KCNMB2-AS1 Diminished Bladder Cancer Cell Stemness

**Figure 3:**
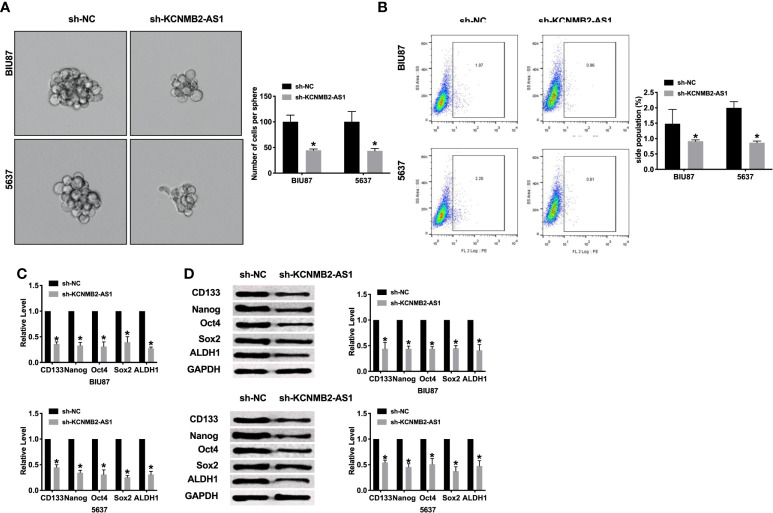
Inhibition of KCNMB2-AS1 suppressed cancer stem cell properties. **(A)** Tumor sphere-forming ability was assessed. n = 6. **(B)** The side population of bladder cells was determined by flow cytometry. n = 5. **(C)** The mRNA level of CD133, Nanog, Oct4, Sox2, and ALDH1 was detected by RT-PCR. n = 10. **(D)** The protein level of CD133, Nanog, Oct4, Sox2, and ALDH1 was detected by western blot. n = 5. *P < 0.05.

Most scholars believe that bladder cancer stem cells are the cause of recurrence and metastasis of bladder cancer. Therefore, a meaningful means to hinder the development of the tumor is to prevent tumor cell stemness. Silencing of KCNMB2-AS1, the sphere-forming ability of BIU87 and 5637 cells was decreased, which was detected by sphere formation assay (**Figure 3A**). Flow cytometry assay performed the decreased side population of bladder cancer cells in the sh-KCNMB2-AS1 transfection group (**Figure 3B**). Then we detected the level of cancer stem cell markers (CD133, Nanog, Oct 4, Sox 2, and ALDH1). Knockdown of KCNMB2-AS1 markedly down-regulated expression of CD133, Nanog, Oct4, Sox2, and ALDH1 (**Figures 3C, D**). Taken together, knockdown of KCNMB2-AS1 could d dwindled bladder cancer cell stemness.

### KCNMB2-AS1 Interacts With miRNA-3194-3p

**Figure 4:**
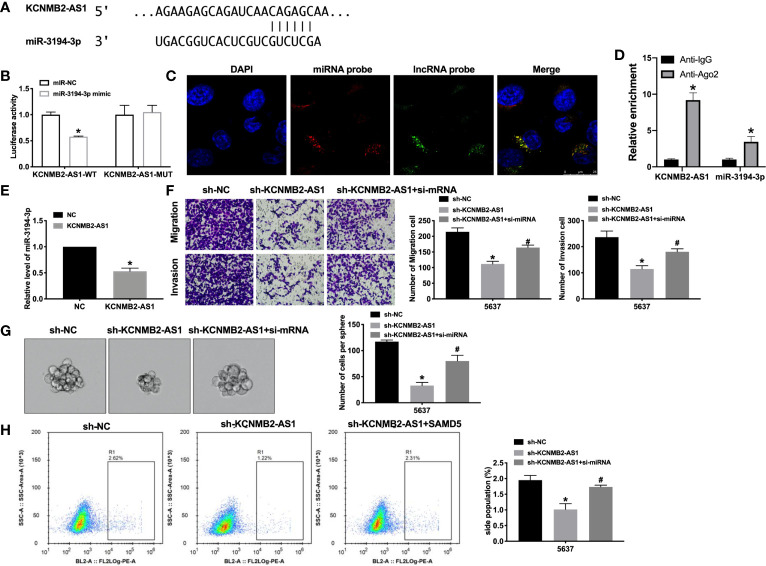
KCNMB2-AS1 sponges to miR-3194-3p. **(A)** miRNA binding sites prediction of lncRNAs in Starbase 3.0. **(B)** The activity of luciferase was measured. n = 3. **(C)** Co-localization of miR-3194-3p and KCNMB2-AS1 by FISH. **(D)** The enrichment analyze by AGO2 RIP assay. **(E)** The expression level of miR-3194-3p was detected. n = 8. **(F)** Detecting cell migration and invasion by Transwell invasion assay. n = 5. **(G)** Tumor sphere-forming ability was assessed. n = 5. **(H)** The side population of bladder cells was determined by flow cytometry. n = 5. *P < 0.05, ^#^ *P* < 0.05.

Previous studies have found that the interaction between microRNA and lncRNA plays a key role in the occurrence and development of tumors. Bioinformatics website predicted the existence of binding sites between KCNMB2-AS1 and miR-3194-3p (**Figure 4A**). Next, we performed luciferase assays to confirm whether KCNMB2-AS1 could bind with miR-3194-3p. The assay report showed that KCNMB2-AS1-WT could bind with miR-3194-3p, which was performed by decreased fluorescence intensity (**Figure 4B**). Further analysis of the data revealed the co-location of KCNMB2-AS1 and miR-3194-3p (**Figures 4C, D**). Further statistical tests revealed that KCNMB2-AS1 could inhibit the expression level of miR-3194-3p (**Figure 4F**). The results, as shown in **Figure 4E**, indicated that silencing of miR-3194-3p would abolish the inhibition of bladder cancer cell migration and invasion by sh-KCNMB2-AS1. Strong evidence was found si-miR-3194-3p would recovery the stemness of bladder cancer (**Figures 4G, H**). These results suggested that miR-3194-3p could be downstream of KCNMB2-AS1 and involve in the regulation of bladder cancer cell development.

### MiR-3194-3p Binds With 3’UTR of SAMD5

**Figure 5:**
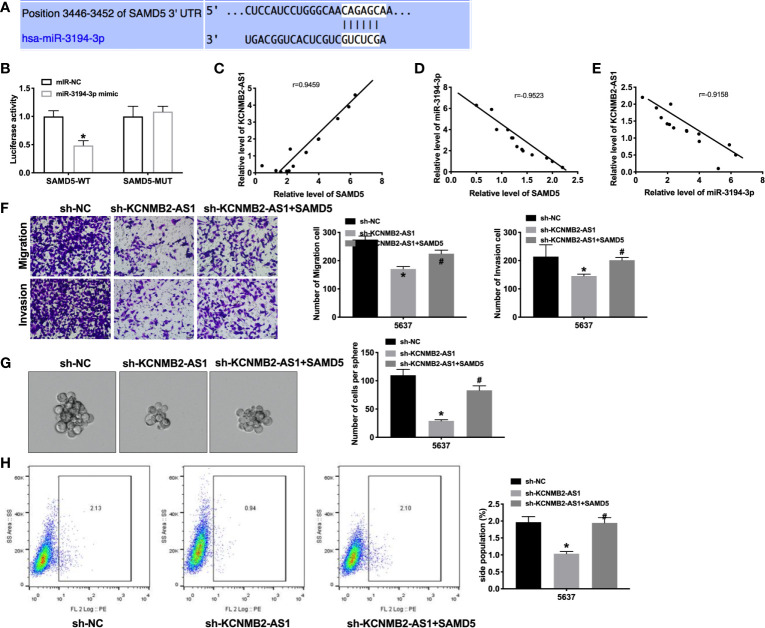
SAMD5 is a target of miR-3194-3p. **(A)** miRNA binding sites prediction of SAMD5. **(B)** The activity of luciferase was measured. n = 3. **(C)** The correlation between KCNMB2-AS1 and SMAD5 expression. n = 14. **(D)** The correlation between SAMD5 and miR-3194-3p expression. n = 14. **(E)** The correlation between KCNMB2-AS1 and miR-3194-3p expression. n = 14. **(F)** Detecting cell migration and invasion by Transwell invasion assay. n = 5. **(G)** Tumor sphere-forming ability was assessed. n = 5. **(H)** The side population of bladder cells was determined by flow cytometry. n = 5. *P < 0.05, ^#^ *P* < 0.05.

Next, we tried to determine the downstream and target of miR-3194-3p. Bioinformatics website predicted the existence of binding sites between miR-3194-3p and SAMD5 (**Figure 5A**). Further analysis of luciferase data revealed that miR-3194-3p could bind with 3’UTR of SAMD5 (**Figure 5B**). There was a significant positive correlation between KCNMB2-AS1 and SAMD5, and a negative correlation between miR-3194-3p and SAMD5, KCNMB2-AS1 and miR-3194-3p (**Figures 5C–E**). As shown in **Figure 5F**, indicated that forced expression of SAMD5 would abolish the inhibition of bladder cancer cell migration and invasion by sh-KCNMB2-AS1. Further evidence was found overexpression of SAMD5 would recovery the stemness of bladder cancer (**Figures 5G, H**). These results suggested that SAMD5 participated in the regulation of bladder cancer cells development by KCNMB2-AS1.

### Knockdown of KCNMB2-AS1 Prevents Tumor Growth *In Vivo*

**Figure 6:**
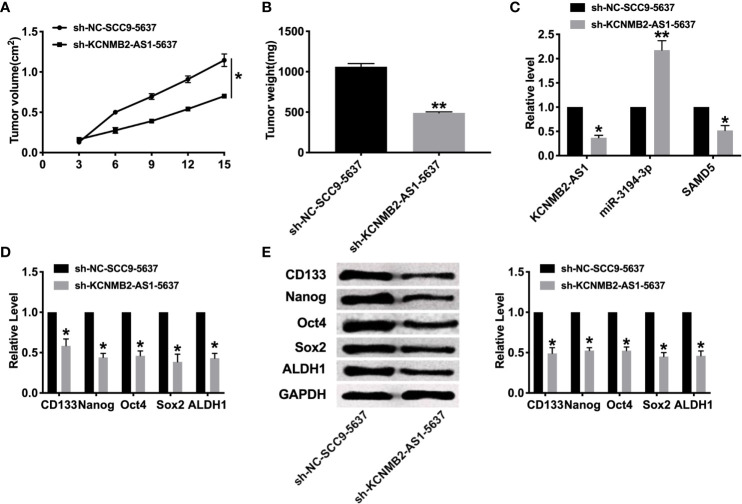
Effect of KCNMB2-AS1 on tumor growth by *in vivo*. **(A)** Tumor volume was measured and calculated. n = 15. **(B)** Effect of KCNMB2-AS1 on tumor growth by *in vivo* tumor formation experiment. n = 10. **(C)** The expression of KCNMB2-AS1, miR-3194-3p, and SAMD5. n = 6. **(D)** The mRNA level of CD133, Nanog, Oct4, Sox2, and ALDH1 was detected by RT-PCR. n = 10. **(E)** The protein level of CD133, Nanog, Oct4, Sox2, and ALDH1 was detected by western blot. n = 6. *P < 0.05, **P < 0.01.

To further explore the function of KCNMB2-AS1 in bladder cancer, we constructed stable low expression of KCNMB2-AS1 5637 cell (sh-KCNMB2-AS1-5637), the normal level of KCNMB2-AS1 5637 cell as a control (sh-NC-5637). Two groups of 5637 cells were randomly and subcutaneously injected in the right lower limb of the nude mice. Then we measured tumor volume. Sh-KCNMB2-AS1-5637 significantly reduced tumor volume and weight (**Figures 6A, B**). Then we detected the expression of KCNMB2-AS1, miR-3194-3p, and SAMD5 (**Figure 6C**). Further, the expression of stem cell markers was assessed by RT-PCR and western blot, the down-regulated level of CD133, Nanog, Oct4, Sox2, and ALDH1 was induced after knockdown of KCNMB2-AS1 (**Figures 6D, E**).

## Discussion

Previous studies have found that the interaction between microRNA and lncRNA plays a crucial function in the occurrence and development of tumors. Here, we observed increased expression of KCNMB2-AS1 in bladder cancer. Knockdown of KCNMB2-AS1 *in vitro* prevented the ability of proliferation, metastasis, and stemness of cancer cells. *In vivo*, silencing of KCNMB2-AS1 also prevented tumor growth. Meanwhile, we revealed that KCNMB2-AS1 could interact with miR-3194-3p and revealed that SAMD5 was a target of miR-3194-3p. In conclusion, our results described that KCNMB2-AS1 would be a novel target of bladder cancer therapy.

LncRNA has been proved that it can participate in the progression of a variety of kinds of cancer. Studies have shown that the expression of HIF1A-AS2 is up-regulated in bladder cancer tissues and cells, and the knockout of IncRNA can inhibit cell proliferation and migration and lead to apoptosis. In addition, high expression of HIF1A-AS2 in healthy human urothelial cells can promote cell proliferation and migration, while inhibit cell apoptosis (21). In bladder cancer, the expression of MALAT1 is up-regulated, and there is a negative correlation between the expression of MALAT1 and E-cadherin (E-cadherin). MALAT1 silencing prevented the epithelial–mesenchymal transformation of (EMT) induced by transforming growth factor β (TGF-β). It is suggested that MALAT1 plays a role in cancer progression and metastasis by enhancing EMT (16). Without affecting cell cycle distribution and apoptosis, TUG1 knockout reduces the ability of proliferation and migration of cancer cells. Recently, it has been reported that TUG1 silencing inhibits the proliferation and leads to apoptosis of bladder cancer cells by inhibiting Wnt/B-catenin pathway (22). LINC00346 silencing can inhibit the proliferation and migration of bladder cancer cells, and trigger cell cycle arrest and apoptosis. In addition, implanting these bladder cancer cells into mice will reduce the tumor growth rate of mice (23). Another study showed that the association between the increased expression of H19 and EZH2 enhancers in patients with bladder cancer caused the stimulation of Wnt/p-catenin pathway, resulting in the down-regulation of E-cadherin, thus promoting the metastasis of bladder cancer cells (24). In addition, the expression of AB074278 is up-regulated in patients with bladder cancer, and may play the role of anti-apoptosis and promoting proliferation by interacting with tumor inhibitory factor and negative regulator of cell proliferation EMP1 (25).

In recent years, with the in-depth study of tumor stem cells, it is considered that tumor stem cells have characteristics similar to normal tissue stem cells, and tumor stem cells have the ability of self-renewal, self-proliferation, and multi-directional differentiation (26). The existence of bladder cancer stem cells plays an important role in maintaining the occurrence, development, proliferation, invasion, and metastasis of bladder cancer. Tumor stem cells have the potential of self-renewal, unlimited proliferation, and multi-directional differentiation (27). In recent years, remarkable achievements have been made in the related research of urinary tumor stem cells. It has been found that bladder cancer stem cells may originate from the mutation of epithelial basal stem cells or the mutation formation of non-stem cell bladder cancer cells. At present, great progress has been made in the research of molecular markers on bladder cancer stem cells surface, such as CD133, Nanog, Oct4, Sox2, and ALDH1. In previous research, lncRNA NCK1-AS1 could promote proliferation and stemness of bladder cancer through remitting the expression level of miR-143 (28). Zhang et al. performed that silencing of lncRNA SOX2OT prevented the stemness phenotype of bladder cancer cells. Moreover, inhibition of SOX2OT remitted xenograft tumor growth and metastases *in vivo* (29). LncRNA XIST interacted with miR-200c and regulated the stemness ability of bladder cancer cells (30). In our research, we found that forced decreased expression of KCNMB2-AS1 would inhibit the expression of CD133, Nanog, Oct4, Sox2, ALDH1 and prevent the ability of stemness of bladder cancer cell lines, which would provide a new target of Bladder cancer treatment.

Limitations, however, still existed in this study. For example, because we examined only 56 patients, our outcomes may be accidental, which needs to be confirmed in a large scale. Besides, the mechanism of KCNMB2-AS1–miR‐3194-3p–SMAD5 axis may have different effects on different stages of bladder cancer, but this has not been further studied in our research. Nevertheless, we deem the trend would be analogical, which can be ascertained in years to come. Jointly, our study served as a proof that KCNMB2-AS1 may act as an oncogenic lncRNA to stimulate the development of cancer through miR‐3194-3p/SMAD5 pathway and enjoy a great possibility to be a therapeutic target and promising biomarker for bladder cancer.

Bladder cancer is a common malignant tumor in the world. Although there are many methods in the diagnosis of bladder cancer at home and abroad in recent years, such as virtual cystoscopy, there are still many problems. In this case, if invasive bladder cancer is not diagnosed and treated early, it will eventually develop into invasive bladder cancer. There is an urgent need to understand the defects of gene regulatory networks at the genome level. Recently, thousands of LncRNA have been identified, and disease-related LncRNA spectra have been obtained by various molecular methods. Functional studies have shown that some LncRNA is involved in the pathogenesis of human cancer and can be used as both oncogenes and tumor suppressors. Some bladder cancer-related LncRNA plays an important biological role in tumorigenesis and metastasis, providing an opportunity to develop new biomarkers for bladder cancer diagnosis and potential for targeted therapy or to become a specific marker for clinical diagnosis of bladder cancer and a theoretical basis for new targets for bladder cancer intervention. The biological mechanism of LncRNA is opening up a new way to unravel the cause, which may place LncRNA in the central stage of cancer biology, open a new door for the diagnosis and treatment of bladder cancer, and provide a new possibility for the realization of sensitive early screening and postoperative detection system of bladder cancer.

## Data Availability Statement

The original contributions presented in the study are included in the article/supplementary material. Further inquiries can be directed to the corresponding author.

## Ethics Statement

The studies involving human participants were reviewed and approved by Harbin Medical University Cancer Hospital. The patients/participants provided their written informed consent to participate in this study. The animal study was reviewed and approved by Harbin Medical University Cancer Hospital.

## Author Contributions

Conceptualization, C-FL. Formal analysis, Y-SC and C-FL. Funding acquisition, Y-SC. Investigation, Y-PX. Methodology, Y-PX. Project administration, W-HL. Resources, W-HL. Software, D-CL. Supervision, D-CL. Validation, HW. Visualization, HW. Writing—original draft, HW. Writing—review and editing, HW. All authors contributed to the article and approved the submitted version.

## Funding

This work is supported by Special Fund Youth Reserve Talent Project for Harbin Science and Technology Innovation Talent Research (NO. 2016RAQXJ150).

## Conflict of Interest

The authors declare that the research was conducted in the absence of any commercial or financial relationships that could be construed as a potential conflict of interest.
